# South American Spider Mites: New Hosts and Localities

**DOI:** 10.1673/031.011.12101

**Published:** 2011-09-18

**Authors:** Renata S Mendonça, Denise Navia, Ivone R Diniz, Carlos HW Flechtmann

**Affiliations:** ^1^ Universidade de Brasília, UNB, Instituto de Ciências Biológicas, Departamento de Zoologia, Campus Universitário Darcy Ribeiro, ICC Sul, Sala AT-159, Asa Norte, 70910-900, Brasília-DF, Brazil; Université Montpellier II, Montpellier, France; ^2^ Embrapa Recursos Genéticos e Biotecnologia, Laboratório de Quarentena Vegetal, Parque Estação Biológica, W5 Norte Final, Caixa Postal 02372, 70.770-900 Brasília-DF, Brazil; ^3^ CNPq Researcher, Escola Superior de Agricultura Luiz de Queiroz (ESALQ), Universidade de São Paulo, Cx. Postal 09, 13.418-900, Piracicaba-SP, Brazil

**Keywords:** Eurytetranychini, Hystrichonychini, neotropical region, systematics, taxonomy, Tetranychini

## Abstract

In order to contribute to taxonomic information on Tetranychid mites (Acari: Tetranychidae) in South America, surveys were conducted in Brazil (15 States and the Federal District) and Uruguay (one Department); 550 samples of 120 plant species were collected. Tetranychid mite infestations were confirmed in 204 samples, and 22 species belonging to seven genera of the Bryobiinae and Tetranychinae subfamilies were identified on 58 different host plants. Thirty-six new plant hosts were found in Brazil, South America, and worldwide for the following species: *Eutetranychus banksi* (McGregor); *Mononychellus tanajoa* (Bondar); *Oligonychus anonae* Paschoal; *O. mangiferus* (Rahman and Sapra); *Tetranychus bastosi* Tuttle, Baker and Sales; *T. desertorum* Banks, 1900, *T. evansi* Baker and Pritchard; *T. ludeni* Zacher; *T. mexicanus* (McGregor); *T. neocaledonicus* André; and *T. urticae* Koch. Four new localities in Brazil were reported for *Eotetranychus tremae* De Leon; *O. anonae*; *Panonychus ulmi* (Koch); and *T. gloveri* Baker and Pritchard.

## Introduction

The Tetranychidae Donnadieu family includes a large number of strictly phytophagous mites; a few species are significant agricultural crop pests worldwide (Jeppson et al. 1975; Helle and Sabelis 1985).

The number of described tetranychids remained stable for 75 years and then increased when their economic incidence for agriculture became more significant (Bolland et al. 1998). In 1950, McGregor initially listed 102 species in 15 genera. Five years later, this number increased to 204 species in 18 genera (Pritchard and Baker 1955). In 1998 there were 1189 species in 71 genera registered in the World Catalogue of the Spider Mite Family (Acari: Tetranychidae) (Bolland et al. 1998), and currently there are approximately 1257 species in 76 genera listed on the Spider Mite Web, which is a comprehensive database on the Tetranychidae (Migeon and Dorkeld 2006).

In Brazil, taxonomic studies on the Tetranychidae were initially conducted between 1920 and 1930. Among the early findings was the report of *Tetranychus gloveri* Banks 1900 and the description of *Mononychellus tanajoa* (Bondar 1938) based on specimens collected in Brazil by G Bondar, a Russian researcher living in the state of Bahia (Bondar 1930, 1938). In the 1960s numerous contributions were made by pioneers in agricultural mite studies in Brazil, such as CHW Flechtmann (Flechtmann 1967, 1967a, 1967b, 1972, 1975, 1976; Flechtmann and Baker 1970, 1975) and AD Paschoal (Paschoal 1970, 1970a), followed by RJF Feres in subsequent decades (Feres 1986, 1992; Feres and Flechtmann 1986, 1986a, 1988, 1995, 1995a, 1995b). From that time forward these and other researchers have conducted major studies on tetranychids in several fields such as taxonomy, ecology, biology, control, and resistance, bringing important advances to knowledge of Brazilian tetranychids. Currently 185 of the 1257 described tetranychid species are reported in South America, of which 104 are found in Brazil (Bolland et al. 1998; Migeon and Dorkeld 2006).

The most intensive search for Tetranychidae mites in Brazil, the largest country in South America, has been centered in the southeast region and is usually associated with economically important crops. Agriculture continues to expand, approachinbiological reserves and other regions in the country. It is important to continue tetranychid research in previously unexplored areas in hopes of increasing our understanding of this family. This paper presents new information about the occurrence and distribution of Tetranychidae in five Brazilian regions and in Uruguay, on plants ranging from ornamental, fruit-bearing, vegetable, grass, oilseed, to large crops species. New hosts and localities for tetranychid mites in Brazil, South America, and the world are reported.

## Materials and Methods

A legal permit to collect zoological material for scientific purposes is required by the ***Brazilian Institute*** for the ***Environment*** and ***Renewable*** Natural Resources (IBAMA). A permit was requested and permit number 231/2006 CGFAU/IBAMA was issued.

### Mite collection

**Figure 1.**
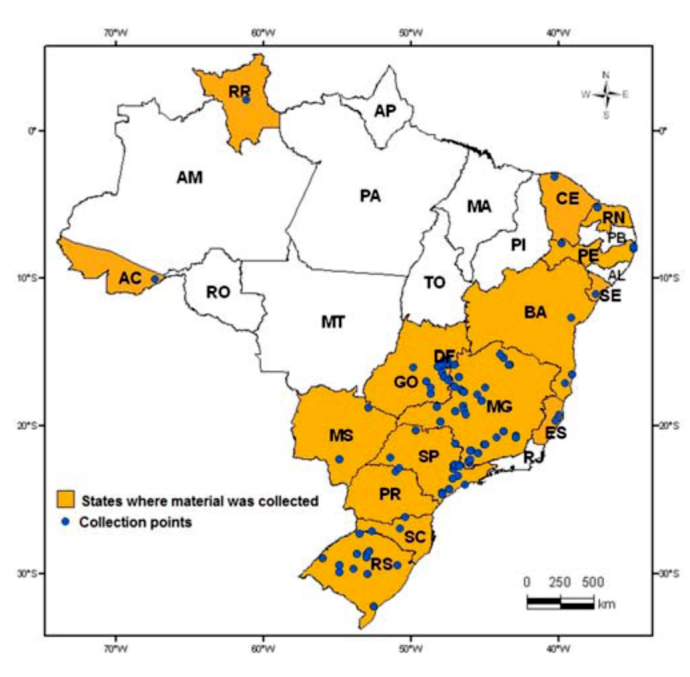
States and their respective sampling points for collecting tetranychid mites in Brazil between October 2004 and July 2008. ARCGIS 9.0 (ESRI, www.esri.com). Latitude and longitude coordinates for the single sample point from Uruguay were 58.1312 S and 32.6368 W. The sample point was not mapped. High quality figures are available online.

Mites were collected between October 2004 and July 2008. A total of 550 samples were collected from several host plants found in five regions in Brazil that include the states of Acre, Bahia, Ceará, Espírito Santo, Goiás, Minas Gerais, Mato Grosso do Sul, Paraná, Pernambuco, Rio Grande do Norte, Rio Grande do Sul, Roraima, Santa Catarina, São Paulo, Sergipe, and the Federal District (Figure 1; Appendix 1). One additional collection was conducted in Uruguay. Plants were collected when symptoms of tetranychid attacks were observed, according to Moraes and Flechtmann (2008). The geographical points of collection were logged into a Garmin 12 GPS using WGS84 (World Geodetic System 84) datum; maps were created using ARCGIS 9.0 (ESRI, www.esri.com).

### Inspecting and processing plant samples

Mites were collected from the plants using a washing/sieving extraction method (Miranda et al. 2007). The resulting 70% ethanol solution was inspected under Stemi SV6 (www.zeiss.com) and Olympus SZX 122 (www.olympus.com) stereomicroscopes connected to a Highlight 3000–1 transilluminator at 50^×^ magnification. Finally, Tetranychidae mites were directly mounted on microscopic slide preparations in Hoyer's medium. From each population, 25 females were mounted in dorso-ventral position and ten isolated males were mounted in lateral position to identify the species by morphological analysis. When only a few males were present in a sample, all of them were slide-mounted.

### Identifying mite species

Microscopic slide preparations of specimens were examined under phase contrast (Leitz Wetzlar, www.leica-microsystems.com) and interference microscopes (Nikon Eclipse 80i, www.nikon.com) in 40× and 100× objectives and. Morphological identification was conducted by examining relevant taxonomic characteristics of male and female Tetranychidae systematics. The shape of the aedeagus, or male genitalia, was used to classify the species (Pritchard and Baker 1955, Meyer 1974, 1987, Baker and Turtle 1994, Ehara, 1999), which were then compared to specimens that had been deposited in the Reference Mite Collection of the Laboratory of Plant Quarantine, Embrapa Genetic Resources and Biotechnology, Brasilia, Brazil. Materials from this study were deposited in this collection as voucher specimens.

## Results and Discussion

Tetranychid mites were found on 37.1% (n = 204) of the 550 plant material samples that were collected (Appendix 1). Twenty-one species of Tetranychinae and one of Bryobiinae were identified on the 204 samples from different localities and inspected hosts (Table 1).

**Table 1.**
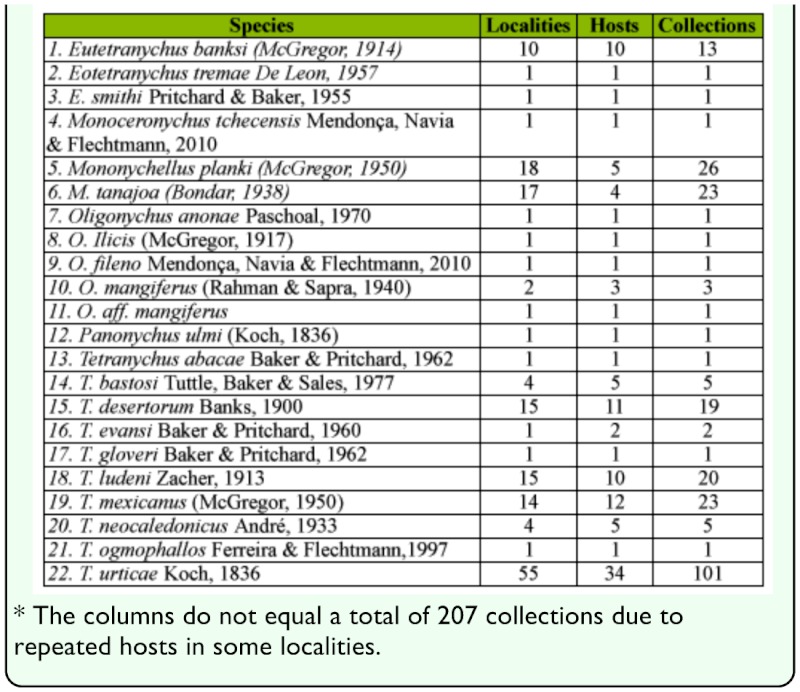
Tetranychid mite species (Tetranychinae) collected in Brazil between October 2004 and July 2008.

Several samples contained more than one species of tetranychid on the same leaf. This occurred on bean (*T. urticae* and *Eutetranychus banksi*; *T. urticae* and *M. planki*; *T. urticae, T. ludeni*, and *T. desertorum*; *T. desertorum* and *M. planki*; *T. ludeni* and *M. planki*), soybean (*T. urticae* and *M. planki*; *T. urticae* and *T. desertorum*; *T. urticae*, *T. desertorum*, and *M. planki*; *T. ludeni* and *M. planki*; *T. desertorum* and *M. planki*), cotton (*T. urticae and M. planki*; *T. ludeni* and *M. planki*; *T. ludeni* and *T. mexicanus*), okra (*T. neocaledonicus* and *M. planki*), and eggplant (*T. urticae* and *M. tanajoa*). *T. mexicanus*, *P. ulmi*, *Oligonychus aff. mangiferus*, and *O. fileno* were found on grapes in the municipality of Pirapora. Foott (1962, 1963) reported phytophagous mite species coexisting on a single host. The fact that different species coexist on a single host requires careful sampling and collecting larger numbers of specimens in microscopic preparations. This allows for greater representation of mite fauna and increases the probability of identifying all Tetranychidae species present on a specific host and/or at a specific locality.

**Table 2.**
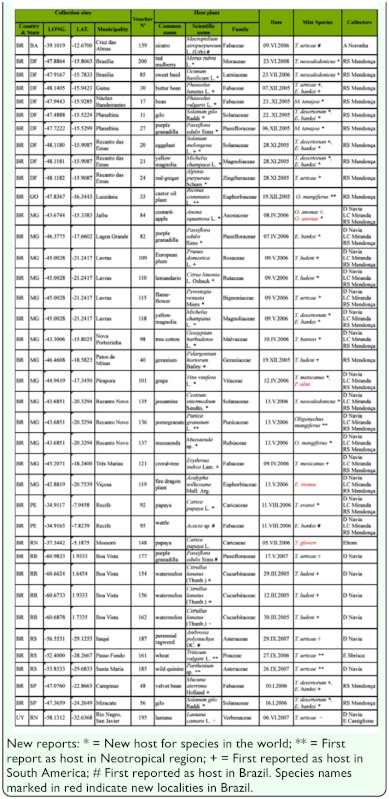
New hosts and localities for tetranychid mites species collected in Brazil and Uruguay between October 2004 and July 2008 with information on collection sites, host plants, collecting dates, and collectors.

### New hosts for Tetranychidae mites in Brazil, South America, and the world

New hosts were found for 11 tetranychid mite species: *E. banksi*, *M. tanajoa*, *O. anonae*, *O.* *mangiferus*, *T. bastosi*, *T. desertorum*, *T. evansi*, *T. ludeni*, *T. mexicanus*, *T. neocaledonicus*, and *T. urticae* (Table 2). The new hosts are listed below according to mite species and host plant family.

Tetranychinae Berlese, Eurytetranychini Reck

### ***Eutetranychus banksi*** (McGregor 1914)

*Tetranychus banksi* McGregor, 1914. Type-host: *Ricinus communis* L. Type-locality: Orlando, Florida, United States.

### Fabaceae

*Acacia* sp., (acacia), Universidade Federal Rural de Pernambuco, UFRPE, Recife, Pernambuco, 11.VIII.2006.

*Mucuna aterrima* Holland (black mucuna), Institute Agronômico de Campinas, ICA, Campinas, São Paulo, 10.I.2006.

*Phaseolus lunatus* L. (lima beans), Embrapa Hortaliças, Gama, Distrito Federal, 07.XII.2005.

The presence of *E. banksi* on *Acacia* sp. was previously reported in Colombia (Urueta 1975). The occurrence on this host from Pernambuco is the first in Brazil.

McGregor (1914) described *E. ban*ksi on *M. pruriens* in the United States and Livshits and Salinas-Croche (1968) located it on *Mucuna* sp. in Cuba. Garret et al. (1967) reported *E. banksi* infestations on *P. lunatus* in Hawaii. This is the first report of *E. banksi* on *M. aterrima* and *P. lunatus* in South America.

### Magnoliaceae

*Michelia champaca* L., (yellow magnolia), Faculdade da Terra, Recanto das Emas, Federal District, 28.XI.2005.

### Passifloraceae

*Passiflora edulis* Sims (sour passion fruit), Lagoa Grande, Minas Gerais, 07.IV.2006.

### Solanaceae

*Solanum gilo* Raddi (scarlet eggplant), Pipiripau, Planaltina, Distrito Federal, 22.XI.2005 and Caturra farm, Vale do Ribeira, Miracatu, São Paulo, 16.I.2006. Caturra farm, Miracatu, Vale do Ribeira, São Paulo, 16.I.2006.

*Solanum melongena* L. (eggplant), Faculdade da Terra, Recanto das Emas, Federal District, 28.XI.2005.

*Michelia champaca*, *P. edulis*, *S. gilo* and *S. melongena* are new hosts for *E. banksi* in the world.

Tetranychinae Berlese, Tetranychini Reck

### ***Mononychellus tanajoa*** (Bondar 1938)

*Tetranychus tanajoa* Bondar, 1938. Type-host: *Manihot ultilissima* and *M. aipim.* Type-locality: Bahia, Brazil.

### Fabaceae

*Phaseolus vulgaris* L. (beans), Vargem Bonita, Núcleo Bandeirantes, Distrito Federal, 23.XI.2005.

This is the first report of *M. tanajoa* infesting beans (*P. vulgaris*) under field conditions. This mite mainly infests species from the *Manihot* genus (Euphorbiaceae) and is also reported on plants from the Asteraceae, Caesalpiniaceae, Curcubitaceae, Passifloraceae, Malvaceae, Rubiaceae, and Solanaceae families in the northeastern region of Brazil (Tuttle et al. 1977; Moraes et al. 1995). Plants of the Fabaceae family were cited as hosts for the *M. tanajoa* in Brazil (*Canavalia Braziliensis* Mart. ex Benth., *Macroptilium martii* Benth) (Moraes et al. 1995), in Central America (*Erythrina* sp., *Gliricidia maculata* Kunth) (Andrews and Poe 1980; Gutierrez 1986; Bolland et al. 1998, Migeon and Dorkeld 2006), and Mexico ([*Gliricidia sepium* (Jacq.) Kunth))] (Tuttle et al. 1976).

### Passifloraceae

*Passiflora edulis* Sims (yellow passion fruit), Faculdades Integradas da União Pioneira de Integração Social, UPIS, Planaltina, Distrito Federal, 06.XII.2005.

*Passiflora edulis* is a new host for *M. tanajoa.* This mite was previously reported on *P. cincinnata* Mart. by Moraes et al. (1995) in the northeast region of Brazil (Moraes et al. 1995). The passion fruit plants (*P. edulis*) and the bean plants (*P. vulgaris*) were near a cassava plantation (*Manihot esculenta* Crantz) that was highly infested with *M. tanajoa.* According to Moraes et al. (1995), high levels of *M. tanajoa* in cassava plantations can cause to the species to be dispersed by the wind to nearby plants and temporarily infest alternative hosts.

### ***Oligonychus anonae*** Paschoal 1970

*Oligonychus anonae* Paschoal, 1970. Type-host: *Annona muricata* L. Type-locality: Jaboticabal, Brazil.

### Annonaceae

*Annona squamosa* L. (sweetsop), in the Jaíba, Mucambinho Project, Minas Gerais, 08.IV.2006.

This species was reported in São Paulo on soursop (*A. muricata*) (Annonaceae) (Paschoal 1970) and was collected from Lauraceae plants (*Persea americana* Mill.,) and Vitaceae (*Vitis vinifera L.*) in Brazil (Paschoal 1970a). *Annona squamosa* (sweetsop) is a new host for the *O. anonae.*

### ***Oligonychus mangiferus***

(Rahman and Sapra 1940)

*Paratetranychus mangiferus* Rahman and Sapra, 1940. Type-host: *Mangifera indica* L. Type-locality: Pakistan.

### Euphorbiaceae

*Ricinus communis* L., (castor oil plant), BR 040, km 40, Luziânia, Goiás, 19.XII.2005.

### Punicaceae

*Punica granatum* L. (pomegranate), Recanto Novo, Minas Gerais, 13.V.2006.

### Rubiaceae

*Mussaenda* sp. (pink mussaenda), Recanto Novo, Minas Gerais 13.V.2006.

This is the first report of *O. mangiferus* on castor oil plants and pomegranates in the Neotropics. This mite was previously found on these host plants in India (Gupta 1976, Gupta and Gupta 1994). *Mussaenda* L. is reported for the first time as a host for *O. mangiferus.*

### ***Tetranychus bastosi***

Tuttle, Baker, and Sales 1977

*Tetranychus* (*Tetranychus*) *bastosi* Tuttle, Baker, and Sales 1977. Type-host: *Morus rubra* L. Type-locality: Crato, Brazil.

### Malvaceae

*Gossypium barbadense* L. (wild cotton), Empresa de Pesquisa Agropecuária de Minas Gerais — EPAMIG, Nova Porteirinha, Minas Gerais, 10.IV.2006.

This is first report of *T. bastosi* on cotton (*G. barbadense*). This species was observed previously on *Malva rotundifolia* L. (Tuttle et al. 1977).

### ***Tetranychus desertorum***

Banks 1900

*Tetranychus desertorum* Banks, 1900. Type-host: *Larrea tridentata* J. M. Coult, *Phacelia crenulata* Torr. ex S. Watson. Type-locality: Mesilla, United States.

### Fabaceae

*Mucuna aterrima* Holland (black mucuna), Institute Agronômico de Campinas, ICA, Campinas, São Paulo, 10.1.2006.

Baker and Pritchard (1962) reported *T. desertorum* on *M. pruriens* in Central America. Therefore, *M. aterrima* is a new registered host in the Fabaceae family for the *T. desertorum* in South America.

### Magnoliaceae

*Michelia champaca* L. (yellow magnolia), Faculdade da Terra de Brasilia, Recanto das Emas, Distrito Federal, 28.XI.2005 and the Universidade Federal de Lavras, Lavras, Minas Gerais, 06.V.2006.

*Michelia champaca* is a new host for *T. desertorum.*

### Solanaceae

*Solanum melongena* L. (eggplant), Faculdade da Terra, Recanto das Emas, Distrito Federal, 28.XI.2005.

*Solanum gilo* Raddi (scarlet eggplant), Pipiripau, Planaltina, Distrito Federal, 22.XI.2005 and Caturra farm, Vale do Ribeira, Miracatu, São Paulo, 16.1.2006. Caturra farm, Miracatu, Vale do Ribeira, São Paulo, 16.I.2006.

This is the first report of *T. desertorum* on eggplant (*S. Melongena*) in South America previously registered in Japan by Ehara (1956). Infestations of *T. desertorum* in Brazil were registered on *Acnistus cauliflorus* (Flechtmann 1967),; *Brugmansia suaveolens* (Furtado et al. 2006),; *Brunfelsia sp*, (Flechtmann 2004),; *Lycopersicon esculentum* (Flechtmann 1967), and *Solanum tuberosum* (Paschoal 1970a).

*Solanum gilo* is reported as a new host for *T. desertorum.*

### ***Tetranychus evansi***

Baker and Pritchard 1960

*Tetranychus evansi* Baker and Pritchard,, 1960. Type-host: *Lycopersicon esculentum* L. Type-locality: Mauritius. (Indian Ocean).

### Caricaceae

*Carica papaya* L. (papaya), Mumbecas farm, Recife, Pernambuco, 11.VIII.2006.

*Tetranychus evansi* was originally described using samples collected from tomato plants (Baker and Pritchard 1960). However, this mite had already been discovered in Brazil by Silva (1954) who described it as *T. marianae* McGregor. Since then it has been reported in the United States (Bolland et al. 1998) and more recently in Africa, Argentina, China, Spain, France, Greece, Israel, Italy, Puerto Rico, Portugal, and Taiwan (Migeon and Dorkeld 2006). *Tetranychus evansi* is currently reported on 93 host species (Migeon and Dorkeld 2006), most of which belong to the Solanaceae family (Bolland et al. 1998; Moraes et al. 1987). Infestations were reported in Europe of this mite across several plant families (Ferragut and Escudero 1999). However, the only reports on the biology of *T. evansi* are for those found on tomato plants. *Carica papaya* is a new host for the *T. evansi.*

### ***Tetranychus ludeni***

Zacher 1913

*Tetranychus ludeni* Zacher, 1913 (Zacher 1913 *apud* Pritchard and Baker 1955). Type-host: *Cucurbita sp., Salvia splendens* Ker Gawl. Type-locality: St. Cloud (near Paris), , France.

### Cucurbitaceae

*Citrullus lanatus* (Thunb.) Matsum and Nakai (watermelon), Embrapa Roraima, Boa Vista, Roraima, 29.III.2005, 12.III.2005 e 30.III.2005.

### Geraniaceae

*Pelargonium hortorum* L. H. Bailey (geranium), Patos de Minas, Minas Gerais, 19.XII.2005.

### Rosaceae

*Prunus domestica* L. (plumb), Universidade Federal de Lavras, Lavras, Minas Gerais, 09.V.2006.

*Tetranychus ludeni* was previously found on *C.* *lanatus*, *P. hortorum* and *P. domestica* in Africa, Central America, (El Salvador), Australia, and India (Meyer and Ryke 1959; Davis 1968; Andrews and Poe 1980; Gutierrez and Schicha 1983; Gupta and Gupta 1994). This is the first report of these hosts in South America.

### Rutaceae

*Citrus limonia* L. (mandarin-lime), Universidade Federal de Lavras, Lavras, Minas Gerais, 09.V.2006.

This is the first report of *Citrus limonia* as a host for *T. ludeni.*

### ***Tetranychus mexicanus***

(McGregor 1950)

*Tetranychus mexicanus* (McGregor 1950). Type-host: *Citrus sinensis* L. Type-locality: Mexico.

### Fabaceae

*Erythrina indica* Lam. (coral tree), Três Marias, Minas Gerais, 09.IV.2006.

This is the first report of *T. mexicanus* infesting the *E. indica* ornamental plant in South America. According to Migeon and Dorkeld (2006), *T. mexicanus* infests these hosts of the Fabaceae family: *Arachis hypogaea* L., *Bauhinia* sp., *Centrosema pubescens* Benth, *Crotalaria retusa* L., *Erythrina poeppigiana* O. F. Cook, and *Phaseolus vulgaris* L.

### Vitaceae

*Vitis vinifera L.* (grapes.), Pirapora, Minas Gerais, 12.IV.2006.

This is the first report of *T. mexicanus* on plants of the Vitaceae family.

### ***Tetranychus neocaledonicus***

André 1933

*Eotetranychus neocaledonicus* André,, 1933. Type-host: *Gossypium* sp. Type-locality: New Caledonia (Southwest Pacific Ocean).

### Lamiaceae

*Ocimum basilicum* L. (basil), Embrapa Recursos Genéticos e Biotecnologia Cenargen, Brasília, Distrito Federal, 23.VII.2006.

### Moraceae

*Morus rubra* L. (mulberry), SQS 202 Sul, Brasília, Distrito Federal, 23.VI.2008

### Solanaceae

*Cestrum intermedium* Sendtn. (night blooming jasmine), Recanto Novo, Minas Gerais, 13.V.2006

*Cestrum intermedium*, *O. basilicum*, and *M. rubra* are new hosts for *T. neocaledonicus* in the world. However, the presence of *T. neocaledonicus* on *Cestrum* sp. has been registered in the state of Rio de Janeiro (Flechtmann 1981). Other plant species belonging to the *Ocimum* and *Morus* genera (*O. sanctum* L., *M. alba* L., *M. australis* Poir, *M. indica* L., *M. nigra L., M.* sp.) were registered as hosts for *T. neocaledonicus* in Cuba, India, Japan, and Thailand (Livshits and Salinas-Croche 1968; Baker 1975; Gupta 1992; Gupta and Gupta 1994; Ehara and Yamaguchi 2001).

### ***Tetranychus urticae***

Koch 1836

*Tetranychus urticae* Koch, 1836. (Koch 1836 *apud* Pritchard and Baker 1955). Type-host: *Urtica sp.* Type-locality: Regensburg, Germany.

### Asteraceae

*Ambrosia polystachya* DC. (ragweed), Itaqui, Rio Grande do Sul, 29.IX.2007.

*Parthenium* sp. (parthenium), Universidade Federal de Santa Maria, Rio Grande do Sul, 26.IX.2007.

Paschoal (1970b) first confirmed the presence of *T. urticae* on *A. trifida* L. in the state of São Paulo. *Ambrosia polystachya* is a new host for *T. urticae* in Brazil and in the world.

Reports of *Tetranychus urticae* on *Parthenium* sp. were made in India (Gupta and Gupta, 1994). In the Americas, the first report of its presence on *Parthenium* sp. occurred in Rio Grande do Sul.

### Bignoniaceae

*Pyrostegia venusta* Miers (flame vine), Universidade Federal de Lavras, Lavras, Minas Gerais, 09.V.2006.

Vargas et al. (1996) described *T. urticae* infestations on *P. ignea* C. Presl. in Costa Rica. This was the first report of *T. urticae* infesting *P. venusta* in the world.

### Fabaceae

*Phaseolus lunatus* L. (lima beans), Embrapa Hortaliças, Gama, Distrito Federal, 07.XII.2005.

*Macroptilium atropurpureum* (L.) (siratro), Caliman farm, Cruz das Almas, Bahia.

Infestations of *T. urticae* on *P. lunatus* were reported in the United States, India, and Thailand (Reeves 1963; Gupta and Gupta 1994; Ho et al. 1997) and on *M. atropurpureum* in Greece (Hatzinikolis 1969). *Phaseolus lunatus and M. atropurpureum* are new hosts for *T. urticae* in South America.

### Passifloraceae

*Passiflora edulis* Sims (yellow passion fruit), Embrapa Roraima, Boa Vista, Roraima, 17.V.2007.

*Tetranychus urticae* on passion fruit (*P. caerulea* L. and *P. edulis*) was reported in Venezuela (Doreste 1968) and Australia (*Passiflora* sp.) (Gutierrez and Schicha 1983). This is the first report of *T. urticae* on passion fruit (*P. edulis*) in Brazil.

### Poaceae

*Triticum aestivum* L. (wheat), Embrapa Trigo, Passo Fundo, Rio Grande do Sul, 27.IX.2006.

The first *T. urticae* infestation of wheat crops (*Triticum* sp.) was registered in Greece (Hatzinikolis 1969). This wheat infestation in Brazil is the first occurrence in the Americas.

### Zingiberaceae

*Alpinia purpurata* K. Schum (alpinia), Faculdade da Terra de Brasília, Recanto das Emas, Distrito Federal, 28.XI.2005.

Cases of *Tetranychus urticae* on Zingiberaceae (*Curcuma longa* L.) were reported in India (Gupta and Gupta 1994). This is the first report of *T. urticae* on alpinia (*A. purpurata*) in the world.

The single sample collected in Uruguay revealed a new host for the two-spotted spider mite (*T. urticae*).

### Verbenaceae

*Lantana camara* L. (wild sage), Rio Negro, San Javier, Uruguay, 06.VI.2007. (-58.1312 S; -32.6368 W - collected by D. Navia and E. Castiglione).

*Tetranychus urticae* infesting *L. camara* was first observed in the United States and in India (Thewke and Enns 1969; Gupta and Gupta 1994); this is the first record in South America.

### New localities for Tetranychidae mites in Brazil

New localities have been registered for *Eotetranychus tremae*, *O. anonae*, *T. gloveri*, *P. ulmi*, and *E. smithi* (Table 2).

Tetranychinae Berlese, Tetranychini Reck

### *Eotetranychus tremae*

De Leon 1957

*Eotetranychus tremae*, De Leon 1957. Type-host: *Trema floridanum.* Type-locality: Coral Gables, United States.

Reports of *Eotetranychus tremae* infesting *Acalypha* sp. ornamental plant have been registered in Viçosa, Minas Gerais. Previous reports in Brazil were registered in the states of Rio de Janeiro (Flechtmann 1981) and São Paulo (Flechtmann 2004; Daud and Feres 2005; Feres et al. 2005).

### ***Oligonychus anonae*** Paschoal 1970

*Oligonychus anonae* Paschoal, 1970. Type-host: *Annona muricata* L., *Persea americana* Mill., *Vitis vinifera* L. Type-locality: Brazil.

Paschoal (1970a, 1970b) described this species from samples collected from soursop (*Annona. muricata*) in the state of São Paulo. Its presence on *A. squamosa* in the municipality of Mocambinho, in northern Minas Gerais, indicates a new locality for this species in Brazil.

### ***Tetranychus gloveri***

Banks 1900

*Tetranychus gloveri* Banks, 1900. Type-host: *Gossypium hirsutum* L. Type-locality: Baton Rouge, United States

*Tetranychus gloveri* was reported on beans in Bahia (Bondar 1930) and on papaya at an (unspecified location) (Migeon and Dorkeld 2006). Reports of its presence on papaya (*Carica. papaya* L.) in the state of Rio Grande do Norte indicate a new locality for this species.

### ***Panonychus ulmi***

(Koch 1836)

*Tetranychus ulmi* Koch, 1836. Koch (1836). Type-host: *Ulmus sp.* Type-locality: Regensburg, Germany.

Reports of the European red mite (*P. ulmi*) on grape vines (*Vitis vinifera L.*) were registered for the first time in Brazil in 2008 when damages caused by these mites were first noticed on crops in Rio Grande do Sul (Ferla and Botton 2008). In this work, a second occurrence of *P. ulmi* infesting grape vines was reported in Pirapora, state of Minas Gerais.

*Panonychus ulmi* was first reported in Brazil by Flechtmann (1967a), who discovered them on apples imported from Argentina. Soon after, Bleicher (1974) reported their presence on apple trees (*Malus domestica* L.) growing in orchards in the south of the country. However, European countries such as France, Austria, Portugal, Italy, and Greece consider the *P. ulmi* a grape vine pest (Rambier 1958; Artofer 1976; Carmona and Dias 1980; Girolami and Mozzi 1983; Papaioannou-Souliotis et al. 1994). Similar reports were also registered in Morocco and the United States (Reeves 1963; Tixier et al. 2003).

Despite reports of *P. ulmi* on apple trees as early as 1967 (Flechtmann, 1976a), grape vines in Brazil were not affected, in contrast to the damages the *P. ulmi* caused to vineyards in Europe. Navia et al. (1998) hypothesized that the mites that attacked grape vines in Europe had different biotypes than those that infested apple trees. Moraes and Flechtmann (2008) emphasized the importance of avoiding introduction of European mites into Brazil, stating the reason this species was not present on grape vines in Brazil was unknown. They suggested the possibility of biological differences between Brazilian and European mites.

The spread of *P. ulmi* infestation on grape vines from Rio Grande do Sul (2005/2006) to Minas Gerais (2006) state could be the result of the two states trading plant propagation material. The hypothesis that European mites were introduced into Brazil cannot be discarded considering that *P. ulmi* were discovered approximately 40 years ago and have only recently been causing damage to grape vines. Ferla and Botton (2008) state that the probable causes for *P. ulmi* dispersion in Brazil are: the proximity of apple tree plantations to vineyards in the south of Brazil; the trading of infested plant material within Brazil and abroad; and unbalanced apple orchards due to excessive application of chemical products, especially non-selective fungicides.

New occurrences of phytophagous mites in South America were recently reported in the literature. For instance, the citrus Hindu mite, *Schizotetranychus hindustanicus* (Hirst) was collected from citrus in the municipality of Boa Vista, State of Roraima (Navia and Marsaro Jr. 2010), and *Eotetranychus smithi* Pritchard & Baker 1955 from roses in Rio Branco, Acre (Mendonça et al. 2010); both states from the Northern region of the country. *Tetranychus roseus* Gutierrez 1969 was first observed in São Paulo in 2007 (Matioli et al. 2008) and its dispersal in the state was reported by Matioli et al (2010).

These new occurrences for mites in Brazil together with the results obtained in this study indicate the importance of intensified surveys on different host plants in the country. Doing this will broaden the understanding of the Tetranychidae family and give light to management and control of these mite species in agricultural systems.

## Conclusions

Thirty-five new hosts were registered for 11 tetranychid species already known in Brazil: *E. banksi* (6 hosts), *M. tanajoa* (2), *O. anonae* (1), *O. mangiferus* (3), *T. bastosi* (1), *T. desertorum* (4), *T. evansi* (1), *T. ludeni* (4), *T. mexicanus* (2), *T. neocaledonicus* (3), and *T. urticae* (8), as well as one in Uruguay for *T. urticae.* New localities were registered in Brazil for *E. tremae*, *O. anonae*, *T. gloveri* and *P. ulmi.*

## Figures and Tables

**Appendix 1.**
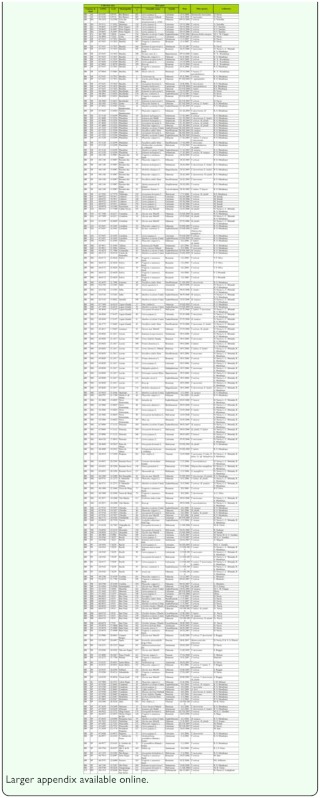
Tetranychid mites species collected in Brazil and Uruguay between October 2004 and July 2008 with information on collection sites, host plants, collecting dates, and collectors.
